# Detection of *Ureaplasma* spp. serovars in genital tract of infertile males

**DOI:** 10.1002/jcla.22865

**Published:** 2019-02-25

**Authors:** Tiejun Song, Zhiwei Liu, Ying Zhang, Yetao Han, Jun Huang

**Affiliations:** ^1^ Department of Clinical Laboratory, Sir Run Run Shaw hospital, School of medicine Zhejiang University Hangzhou China

**Keywords:** infertile males, real‐time PCR, serovars, *Ureaplasma parvum*, *Ureaplasma urealyticum*

## Abstract

**Background:**

The colonization of *Ureaplasma* species in genital tract is related with male infertility. However, it has been postulated based upon limited study that virulence is related to serotype specificity. The aim of this study was to determine the distribution of *Ureaplasma* serovars in genital tract of infertile males and analyze their role in male infertility.

**Methods:**

A total of 358 urethral swabs samples were obtained from infertile males. The culture of *Ureaplasma* species were performed using a commercially available Mycoplasma IST 2 kit. Serovars were determined by real‐time polymerase chain reaction (real‐time PCR).

**Results:**

A total of 92 (25.7%) infertile males were positive for* Ureaplasma* spp; among them, *Ureaplasma parvum* (UPA) was detected in 73 (79.3%) isolates, and *Ureaplasma urealyticum *(UUR) was detected in 19 (20.7%) isolates. Serovars 1, 6, or in combination accounted for 63.0% (46/73) of UPA isolates. Serovar 9 (alone and in combination of other serovars) was the most common serovar in UUR (47.4%, 9/19). Multiple serovars were detected in 21 (22.8%) isolates, and serovars 4, 5, 7, and 12 were not detected in any sample.

**Conclusion:**

The distribution of 14 *Ureaplasma* serovars in genital tract of infertile males was identified for the first time by real‐time PCR assay. UPA serovars 1 and 6, and UUR serovar 9 are the most common serovars colonization in urogenital tract of infertile males.

**Abbreviations**
real‐time PCR: Real‐time polymerase chain reaction
UPA: *Ureaplasma parvum*
UUR: *Ureaplasma urealyticum*

## 1 INTRODUCTION

*Ureaplasma* species belong to the class Mollicutes, bacteria that lack a cell wall, and have been known for decades to colonize the human urogenital tract. There are two species that exist in humans, *Ureaplasma parvum* (UPA) contains serovars 1, 3, 6, and 14 and* Ureaplasma urealyticum* (UUR) contains serovars 2, 4, 5, 7, 8, 9, 10, 11, 12, and 13.^1^, ^2^ About 15%‐30% of healthy adult men may harbor *Ureaplasma* spp in their lower urogenital tract.^1^, ^3^, ^4^ During the past decade, many investigators reported that *Ureaplasma* spp. infection may alter various parameters of the semen, thereby influencing fertilization or pregnancy rates.^5^, ^6^, ^7^, ^8^, ^9^ Most of the previous reports have discussed the role of *Ureaplasma* spp. in male infertility discriminating between the two species. However, few studies concern the influence of particular serovar and their relationship with the male infertility. The aim of this study was to determine the distribution of *Ureaplasma* serovars in genital tract of infertile males, and we have analyzed the role of Ureaplasma serovars in male infertility by comparing results with other studies.

## 2 MATERIALS AND METHODS

A total of 358 males, aged 24‐39 (mean31) years, selected from infertile couples attended the fertility clinic at Sir Run Run Shaw hospital (Zhejiang CN) from January 2013 to October 2013 were enrolled to participate in the present study. All patients had no pregnancy after more than one year of unprotected regular intercourse. The infertility caused by female partners and males who were symptomatic for any genital tract infections in the previous two weeks were excluded from the study. Genital sample collection and culture for *Ureaplasma* spp were performed according to our previous study.^4^ The culture of *Ureaplasma* species was performed by using a commercially available Mycoplasma IST 2 kit (bioMérieux, Marcy L'etoile, France). Genomic DNA was extracted by the proteinase K method as described in our previous study.^10^ Briefly, a total of 0.5 mL of *Ureaplasma* spp. broth culture of each *Ureaplasma* spp. strain was harvested by centrifugation at 12 000* g* for 10 minutes. The cell was resuspended in 50 μL of lysis buffer (10 mmol/L Tris–HCl, pH 8.0; 50 mmol/L KCl; 2.5 mmol/L MgCl_2_; and 0.5% Tween 20) and proteinase K (10 mg/mL), and incubated at 55°C for 1 hour. Then, the sample was heated at 95°C for 10 minutes and centrifuged at 10 000 *g* for 1 minute to remove debris. The supernatant was utilized immediately or stored a −80°C for future use. To distinguish UPA from UUR in isolates of urethral swabs samples, two primer pairs UMS‐125 (GTATTTGCAATCTTTATATGTTTTCG), UMA226 (CAG CTGATGTAAGTGCAGCATTAAATTC), and UMS‐51 (CTGAGCTAT GACATTAGGTGTTACC), UMA 427 (ACCTGGTTGTGTAGTTTCAAAGTTCAC) were used as described by Teng et al^11^ Amplifications were carried out according to the Taq DNA Polymerases (Takara, Japan) protocol as described in our previous study.^10^ UPA and UUR were then typed for their corresponding serovars by a series of serovar‐specific real‐time PCR assays, using the Roche LightCycler 2.0.^12^ Each primer or probe set was confirmed to amplify only the designated serovar in the optimized PCR conditions. The 14 reference strains of the *Ureaplasma* spp. serovars were obtained from the American Type Culture Collection (ATCC), described as follows: ATCC 27813 (UPA serovar 1), ATCC 27814 (UUR serovar 2), ATCC 27815 (UPA serovar 3), ATCC 27816 (UUR serovar 4), ATCC 27817 (UUR serovar 5), ATCC 27818 (UPA serovar 6), ATCC 27819 (UUR serovar 7), ATCC 27618 (UUR serovar 8), ATCC 33175 (UUR serovar 9), ATCC 33699 (UUR serovar 10), ATCC 33695 (UUR serovar 11), ATCC 33696 (UUR serovar 12), ATCC 33698 (UUR serovar 13), and ATCC 33697 (UPA serovar 14).UPA (serovar 3, ATCC 27815) and UUR (serovar 10, ATCC 33699) were used as the quality controls in species‐specific PCR assays. All primers were synthesized by Invitrogen (Shanghai, CN), and probes were ordered by Roche Diagnostics (Shanghai, China). A designated reference strain control and a negative control (distilled water) were included in every PCR run.

## 3 RESULTS

**Figure 1:**
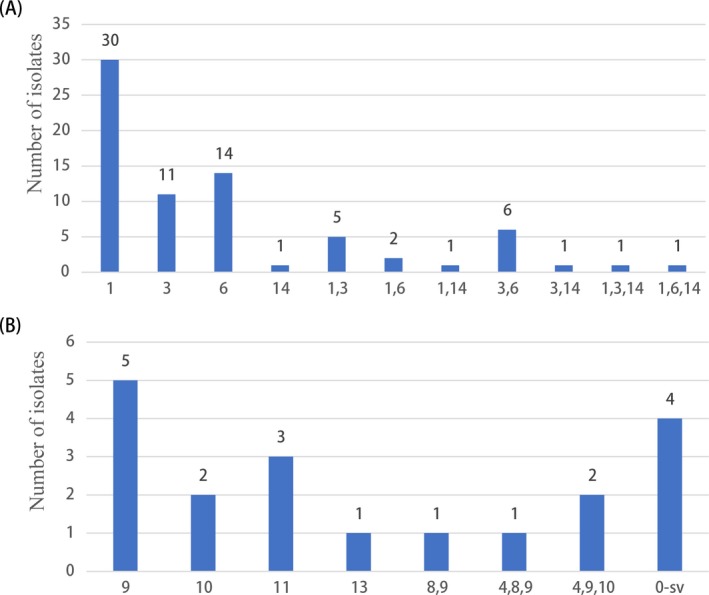
Diversity of serovars among infertile males harbored UPA (A) and UUR (B). 0‐sv, cannot type

A total of 92 (25.7%) males harbored *Ureaplasma* spp. in genital tract among 358 infertile males, UPA was detected in 73 (79.3%) isolates, and UUR was detected in 19 (20.7%) isolates. Serovar genotyping was performed for isolates recovered from 92 samples. As shown in Figure 1A, serovars 1, 6, and in combination accounted for 63.0% (46/73) of UPA isolates. Serovar 14 was detected in five isolates from infertile men alone and in combination with serovar 1, 3, and 6. Serovar 9 (alone and in combination) was the most common serovar in UUR (47.4%, 9/19). Serovars 4, 5, 7, and 12 were not detected in any sample. There were 4 (21.1%, 4/19) isolates that could not be assigned to any of serovars in UUR by real‐time PCR, as shown in Figure 1B. Multiple serovars were detected in 21 (22.8%) isolates, among them, 16 (17.4%) isolates contained two serovars, and 5 (5.4%) isolates contained three serovars.

## 4 DISCUSSION

During the past decades,the role of *Ureaplasma* spp. in male infertility has been controversial. Some investigators failed to show any significant difference in the fertilizing ability of the sperm with a positive culture or any influence on pregnancy rates or outcome,^13^, ^14^, ^15^, ^16^ while others reported that *Ureaplasma* spp. infection may negatively influence semen quality, for example, concentration, activity, motility and/or morphology.^5^, ^6^, ^7^, ^8^, ^9^, ^17^, ^18^ Due to their differential pathogenicity, effort has gone into assignment of isolates into serovars and attempting to correlate specific serovars with specific clinical outcomes, while the ability to differentiate the *Ureaplasma *spp. serovars in clinical samples presented a great challenge for investigators. Previous antibody‐based phenotyping methods yielded inconclusive results because of multiple cross‐reactions and poor discriminating capacity. Later, PCR‐based assays were capable of distinguishing the 4 serovars of UPA, while only divided the 10 serovars of UUR into different subgroups due to limited sequence variation in the PCR targets.^3^, ^19^, ^20^ Until recently, Xiao et al developed a real‐time PCR assays that can separate all 14 ATCC serovars type strains without cross‐reactions.^12^ In this study, we reported the frequency of the all 14 serovars in genital tract of infertile males for the first time using real‐time PCR assays.

Our study indicates that UPA was the predominant species (79.3%) detected among infertile males. This result is in agreement with the result reported by Abusarah et al^21^ and Knox et al,^22^ while it is in contrast to other study, which found UUR to be more common among infertile men.^23^ As to particular serovar and their relationship with the male infertility has been examined in only a few studies.^24^ As shown in Figure 1, we found the most common serovars alone and in combinations with other serovars were UPA serovars 1 and 6, and UUR serovar 9. This is contrary to the previous view that the predominance of *Ureaplasma* spp. serovar 3/14 suggests their possible pathogenic role in genital tract infections and infertility.^24^ They only divided the four serovars of UPA into three subgroups (serovar 1, 6, and 3/14), while incapable of distinguishing the 10 serovars of UUR. Small samples (only 24 isolates) size and serovar 3 and 14 as one subgroup may contribute to this difference. Compared with serotypes of 169 vaginal swabs from healthy pregnant females in previous study.^25^ The results indicated that serovar 1, alone and in combination, was significantly increased in infertile males (40/92, 44%) compared with healthy pregnant females (33/169, 20%); serovar 3, alone and in combination, was significantly decreased in infertile males (26% vs 51%); but serovar 6 and 9 showed no significant difference. However, whether the difference is caused by regional factors or pathogenicity differences still needs to be further verified. In the present study, multiple serovars were detected in 21 (22.8%) isolates, while 21.1% isolates could not be assigned to any serovar in UUR. To determine whether those untypeable isolates represent new serovars or loss of markers needs further investigation.

In conclusion, the distribution of 14 *Ureaplasma* serovars in genital tract of infertile males was described for the first time in China. UPA serovars 1 and 6, and UUR serovar 9 are the most common serovars identified in urogenital tract of infertile males.

## INFORMED CONSENT

All recruited patients provided written informed consent prior to data collection.

## ETHICAL APPROVAL

All procedures were evaluated and approved by the Institutional Ethics Committee of Sir Run Run Shaw Hospital School of Medicine, Zhejiang University.
